# Relationship between adiponectin and leptin on osteocalcin in obese adolescents during weight loss therapy

**DOI:** 10.20945/2359-3997000000039

**Published:** 2018-05-07

**Authors:** Raquel Munhoz da Silveira Campos, Deborah Cristina Landi Masquio, Flávia Campos Corgosinho, Joana Pereira de Carvalho-Ferreira, Bárbara Dal Molin, Ana Paula Grotti Clemente, Lian Tock, Sergio Tufik, Marco Túlio de Mello, Ana Raimunda Dâmaso

**Affiliations:** 1 Universidade Federal de São Carlos Universidade Federal de São Carlos Laboratório de Recursos Terapêuticos Departamento de Fisioterapia São Carlos SP Brasil Departamento de Fisioterapia, Laboratório de Recursos Terapêuticos, Universidade Federal de São Carlos (UFSCar), São Carlos, SP, Brasil; 2 Centro Universitário São Camilo Centro Universitário São Camilo São Paulo SP Brasil Centro Universitário São Camilo, São Paulo, SP, Brasil; 3 Universidade Federal de Goiás Universidade Federal de Goiás Goiânia GO Brasil Universidade Federal de Goiás (UFG), Goiânia, GO, Brasil; 4 Universidade Federal de São Paulo Universidade Federal de São Paulo Programa de Pós-Graduação Interdisciplinar em Ciências da Saúde Santos SP Brasil Programa de Pós-Graduação Interdisciplinar em Ciências da Saúde, Universidade Federal de São Paulo (Unifesp), Santos, SP, Brasil; 5 Universidade Federal de São Paulo Universidade Federal de São Paulo Programa de Pós-Graduação em Nutrição São Paulo SP Brasil Programa de Pós-Graduação em Nutrição, Universidade Federal de São Paulo (Unifesp), São Paulo, SP, Brasil; 6 Universidade Federal de Alagoas Universidade Federal de Alagoas Maceió AL Brasil Universidade Federal de Alagoas (Ufal), Maceió, AL, Brasil; 7 Brasil Weight Science, São Paulo, SP, Brasil; 8 Universidade Federal de São Paulo Universidade Federal de São Paulo Departamento de Psicobiologia São Paulo SP Brasil Departamento de Psicobiologia, Universidade Federal de São Paulo (Unifesp), São Paulo, SP, Brasil; 9 Universidade Federal de Minas Gerais Universidade Federal de Minas Gerais Escola de Educação Física, Fisioterapia e Terapia Ocupacional Belo Horizonte MG Brasil Escola de Educação Física, Fisioterapia e Terapia Ocupacional, Universidade Federal de Minas Gerais (UFMG), Belo Horizonte, MG, Brasil

**Keywords:** Inflammation, bone turnover markers, obesity, adolescents, weight loss

## Abstract

**Objectives:**

Obesity is a multifactorial disease characterized by the presence of the pro-inflammatory state associated with the development of many comorbidities, including bone turnover marker alterations. This study aimed to investigate the role of the inflammatory state on bone turnover markers in obese adolescents undergoing interdisciplinary weight loss treatment for one year.

**Subjects and methods:**

Thirty four post-pubescent obese adolescents with primary obesity, a body mass index (BMI) greater than > 95^th^ percentile of the CDC reference growth charts, participated in the present investigation. Measurements of body composition, bone turnover markers, inflammatory biomarkers and visceral and subcutaneous fat were taken. Adolescents were submitted to one year of interdisciplinary treatment (clinical approach, physical exercise, physiotherapy intervention, nutritional and psychological counseling).

**Results:**

Reduction in body mass, body fat mass, visceral and subcutaneous fat, as well as, an increase in the body lean mass and bone mineral content was observed. An improvement in inflammatory markers was seen with an increase in adiponectin, adiponectin/leptin ratio and inteleukin-15. Moreover, a positive correlation between the adiponectin/leptin ratio and osteocalcin was demonstrated. Further, both lean and body fat mass were predictors of osteocalcin. Negative associations between leptin with osteocalcin, adiponectin with Beta CTX-collagen, and visceral fat with adiponectin were observed.

**Conclusions:**

It is possible to conclude that the inflammatory state can negatively influence the bone turnover markers in obese adolescents. In addition, the interdisciplinary weight loss treatment improved the inflammatory state and body composition in obese adolescents. Therefore, the present findings should be considered in clinical practice.

## INTRODUCTION

Obesity is a multifactorial disease associated with a pro-inflammatory state including a lower adiponectinemia and hyperleptinemia framework. Many comorbidities are associated with obesity, including bone mineral alterations, metabolic syndrome, cardiovascular complications, non-alchoolic fatty liver disease, sleep disorders and asthma (1,2).

Concomitant with this, body fat distributions, especially visceral adipocytes, are linked to the secretion of pro-inflammatory adipokines that can act negatively on bone metabolism (3). In a previous study, it was shown that visceral fat, as well as the visceral/subcutaneous ratio, were independent, negative predictors of bone mineral density (BMD) (4).

Beta-CTx collagen and osteocalcin are bone metabolism biomarkers that may represent bone turnover. Beta-CTx collagen is the C-terminal telopeptide of type I collagen, the main component (approximately 90%) of the protein matrix of bone. Beta-CTx collagen is released into the bloodstream during bone resorption and is almost entirely excreted by the kidneys. Its quantification serves as a specific marker for the degradation of mature type I collagen from bone (5). Osteocalcin is the major noncollagenous protein that acts locally in the bone mineralization. It is synthesized by the osteoblasts and has been utilized as a marker of bone formation or bone turnover (6).

Leptin, an adipokine that is primarily expressed by adipose tissue, is considered to be involved in neuroendocrine control of energy balance. However, in human obesity, hyperleptinemia was associated with a reduction of bone formation biomarkers, especially osteocalcin (7). On the other hand, adiponectin seems to improve bone formation (8), promoting the proliferation, differentiation, and mineralization of osteoblastic cells (9). In addition, serum osteocalcin levels were significantly associated with plasma adiponectin levels and inversely related to leptin levels, in the presence of metabolic syndrome (10).

The adiponectin/leptin ratio is associated as a better inflammatory biomarker of inflammation in metabolic syndrome patients than these adipokines analyzed in isolation (11). However, the relationship between the adiponectin/leptin ratio and bone biomarkers, mostly considering its role in osteocalcin in obese adolescents, has yet to be explored. Moreover, considering obesity to be a multifactorial disease, clinical strategies to promote weight loss, associated with physical exercise, nutrition and psychological interventions can be an interesting approach to promote weight loss and health benefits (12). In this way, the aim of the present investigation is to analyze the effects of interdisciplinary weight loss therapy on pro/anti-inflammatory adipokines and their role in bone turnover markers in obese adolescents.

## SUBJECTS AND METHODS

### Population

For this study, it was involved 34 post-puberty obese adolescents of both genders, with age of 15-19 years. Inclusion criteria were Tanner stage five (13), primary obesity, body mass index BMI > 95^th^ percentile of the CDC reference growth charts (14). Non-inclusion criteria were the use of birth control pills, cortisone, anti-epileptic drugs, history of renal disease, alcohol intake, smoking and secondary obesity due endocrine disorders. There were no obese adolescents with diagnoses of ferritin alteration, autoimmune diseases and virus of Hepatitis A, B and C.

The main reasons for dropping out (n = 4) in our study were financial and family problems, followed by school and job opportunities. No sex differences were observed in adherence rates. The study was conducted with the principles of the Declaration of Helsinki and was approved by the ethics committee on research at the *Universidade Federal de São Paulo* (Unifesp) (0135/04; 152.281), Clinical Trial: NCT01358773. All procedures were clear to those responsible for the volunteers and it was obtained consent for research. All evaluations were performed at two different times (baseline and after interdisciplinary intervention).

### Anthropometric measurements

Weight was measured by plethysmography scale (BODPOD equipment), where patients wore minimum clothing possible and height was measured using a stadiometer (Sanny – model ES 2030). After obtaining the data was calculated using the body mass index (BMI) by dividing the weight by height squared (kg/m²). Body composition, including body fat mass (percentage and kilograms) and body lean mass (percentage and kilograms), was obtained through air displacement pletismography (BODPOD).

### Bone mineral density (BMD) and bone mineral content (BMC)

A whole-body DXA absorptiometry scan was performed per unit of bone densitometry to determine the whole-body of bone mineral density-BMD (g/cm²) and bone mineral content-BMC (grams) using a Lunar Prodigy Advance System (GE Healthcare). The whole-body scan required the subjects to be placed in a supine position with their arms and legs positioned according to the manufacturers’ specifications (15). Quality control was performed daily using a phantom, and measurements were maintained within the manufactures standards of ≤ 1%. In order to obtain statistically precise measurements, 68% of the exams were repeated within a coefficient of variation of 1DP (± 0.010 g/cm³ for total body size).

### Serum analysis

Blood samples were collected at the outpatient clinic at approximately 8:00 A.M. after an overnight fast (12 hours). The adipokines concentrations (adiponectin, leptin and interleukin-15) were measured using a commercially available multiplex assay (EMD Millipore: HMHMAG-34K; HCYTOMAG-60K). Manufacture-supplied controls were included to measure assay variation and all samples were analyzed on the same day to minimize day-to-day variation. A minimum of 100 beads were collected for each analyzed using a Luminex MagPix System (Austin, Texas), which was calibrated and verified prior to sample analysis. Unknown sample values were calculated offline using Milliplex Analyst Software (EMD Millipore) (16). Serum osteocalcin and Beta CTx-collagen levels were obtained using the ECLIA (Electrochemiluminescent immune Assay). For the leptin hormone, the following values were adopted: males, between 1 and 20 ng/mL and females, between 4.9 and 24 ng/mL previously described by Gutin and cols. (17).

### Visceral and subcutaneous adiposity measurements

The abdominal ultrasonography procedures and the measurements of visceral and subcutaneous fat tissue and fatty liver were performed by the same physician, who was blinded to the subject assignment groups at the baseline time-point and following 1 year therapy. This physician was a specialist in imaging diagnostics. A 3.5-MHz multifrequency transducer (broad band) was used to reduce the risk of misclassification. The intra-examination coefficient of the variation for ultrasound (US) was 0.8%. US measurements of intra-abdominal (visceral) and subcutaneous fat were obtained. US determined subcutaneous fat was defined as the distance between the skin and external face of the rectus abdominal muscle, and visceral fat was defined as the distance between the internal face of the same muscle and the anterior wall of the aorta. The cut-off points for the definition of visceral obesity by ultrasonography were based on the previous methodological descriptions made by Ribeiro-Filho and cols. (18).

### Descriptive methodology of interdisciplinary weight loss therapy

**Figure 1:**
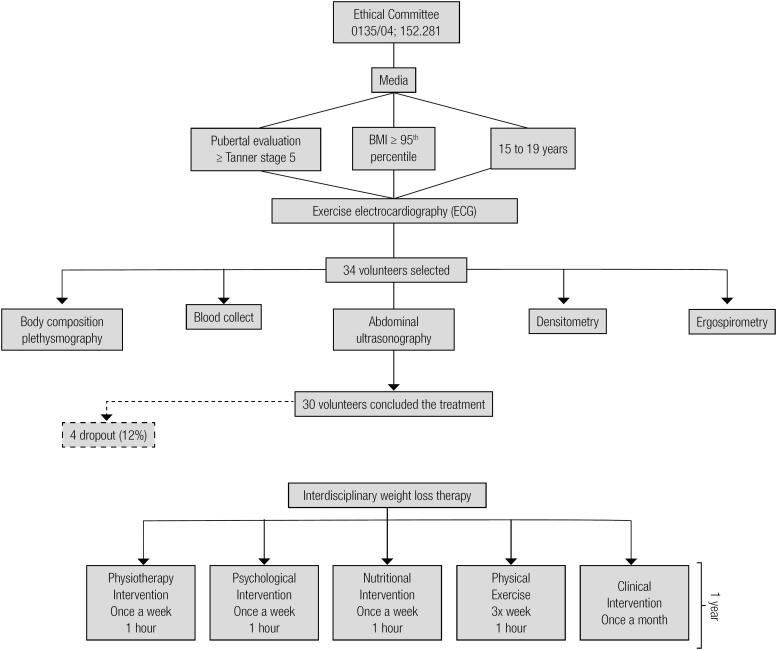
Descriptive methodology of study.

All sessions were conducted by an interdisciplinary group of health professionals. Three times of week during two hours per day not consecutive, the adolescents participated of supervised therapy in physical exercise, nutrition and psychological attendances during one year. Once each month the adolescents were followed by an endocrinologist (Figure 1).

#### Clinical intervention

All obese adolescents visited the endocrinologist with their parents once each month. In all of these visits, the entire GEO team (Study Group of Obesity) was also present. The doctor monitored and evaluated all clinical exams of adolescents and treated health problems during therapy. The medical follow-up included the initial medical history and a physical examination of blood pressure, cardiac frequency and body mass, and the adolescents were checked for their adherence to all interdisciplinary therapies. The team discussed with the adolescents and their parents some possible changes in lifestyle to promote their health (Figure 1).

#### Physical exercise intervention: aerobic plus resistance training (AT + RT)

During the 1-year therapy period, the adolescents followed a combined exercise training therapy. The protocol was performed three times per week for 1 year and included 30 min of aerobic training plus 30 min of resistance training per session. The subjects were instructed to reverse the order of the exercises (aerobic and resistance) at each training session. The aerobic training consisted of running on a motor-driven treadmill (Life Fitness – model TR 9700HR) or bicycle at a cardiac frequency intensity representing the ventilatory threshold I (±4 bpm), which was determined by the results of an initial oxygen uptake test for aerobic exercises (ergoespirometry). The exercise therapy was based on the guidelines from the American College of Sports Medicine (ACSM). Resistance training was also designed based on ACSM recommendations (19,20) (Figure 1).

#### Nutrition intervention

Energy intake was set at the levels recommended by the dietary reference intake for subjects with low levels of physical activity and of the same age and gender following a balanced diet (21). No pharmacotherapies or antioxidants were recommended. Once a week, adolescents had dietetics lessons educating participants on the food pyramid, diet record assessment, weight loss diets and fad diets, food labels, dietetics, fat-free and low-calorie foods and other related topics. Monthly, they had individual consultations (Figure 1).

#### Physiotherapy intervention

The adolescents were accompanied by a physiotherapist during the therapy, in order to prevent musculoskeletal injuries. Once a week, the volunteers had lessons regarding such topics as the postural orientation, prevention of musculoskeletal injuries, diaphragmatic breathing, hydrotherapy, isostretching, and balance.

#### Psychological intervention

Psychological therapy treatment plans were established on the basis of validated questionnaires that considered some of the psychological problems caused by obesity, as described in the literature. These include depression, eating disorders, anxiety, decreased self-esteem and body image disorders. Interdisciplinary therapy consisted of a weekly 1h group session. Individualized psychological therapy was recommended when it was necessary according psychological assessment (Figure 1).

### Statistical analysis

Statistical analysis was performed using the program STATISTICA version 7.0 for Windows Vista. The adopted significant value was α ≤ 5%. Data normality was verified with the Shapiro Wilk test. Parametric data were expressed as mean ± SD, and non-parametric data were expressed as median, minimum and maximum values. The effects of interdisciplinary therapy during 1 year were analyzed by *t* test dependent by samples. For the non-parametric data the Wilcoxon test was applied. Correlations were established through the Pearson test for parametric data and Spearman for nonparametric data. Finally, it was verified the dependencies of variables by simple linear regression. The correlations and regression test were analyzed with baseline and final values.

## RESULTS

The present study was composed by 34 obese adolescents: 18 girls (11 ± 1age of menarche) and 16 boys with 16 ± 1 years old, primary obesity (diagnosis of obesity around 10 ± 3 years old), body mass 94.5 ± 15.45 (kg) and body mass index 33.21 ± 3 (kg/m²).

### Effects of interdisciplinary weight loss therapy

One year of interdisciplinary weight loss therapy demonstrated significantly reduction in body mass (kg), BMI (kg/m²), body fat mass (kg and %), visceral fat (cm), subcutaneous fat (cm) and visceral/subcutaneous ratio. Increases in the values of body lean mass (%), bone mineral content (g), adiponectin (µg/L), adiponectin/leptin ratio, and interleukin-15 (IL-15) (pg/mL) were observed (Tables 1 and 2).

**Table 1:** Effects of interdisciplinary weight loss therapy in body composition of obese adolescents

Variables	Baseline	1 year	*p* value	Δ value
Body mass (kg)	94.50 ± 15.45	88.70 ± 14.62*	< 0.001	-5.27 ± 4.5
Height (m)	1.68 ± 0.10	1.69 ± 0.12	0.60	0 ± 0.002
Body mass index (kg/m²)	33.21 ± 3.09	31.52 ± 3.19*	< 0.001	-1.90 ± 1.61
Body fat mass (%)	40.40 ± 6.50	37.35 ± 7.03*	< 0.001	-3.31 ± 2.98
Body fat mass (kg)	38.19 ± 8.75	33.17 ± 8.32*	< 0.001	-4.98 ± 4.22
Body lean mass (%)	59.60 ± 6.50	62.65 ± 7.03*	< 0.001	3.32 ± 2.98
Body lean mass (kg)	56.38 ± 10.89	57.55 ± 10.84	0.36	1.17 ± 2.00
Bone mineral density (g/cm²)	1.25 ± 0.08	1.26 ± 0.08	0.82	0 ± 0.02
Bone mineral content (g)	3192.21 ± 465.89	3338.12 ± 547.06*	< 0.001	140.34 ± 121.74
Visceral fat (cm)	4.46 ± 1.34	3.69 ± 1.20*	< 0.001	-0.66 ± 0.87
Subcutaneous fat (cm)	3.66 ± 0.79	3.40 ± 0.84*	0.01	-0.44 ± 0.54
Visceral/subcutaneous ratio	1.27 ± 0.45	0.29 ± 1.46*	0.001	-0.91 ± 1.20

* Statistical difference p ≤ 0.05. Effects of therapy: comparison between baseline and 1 year of therapy.

**Table 2:** Effects of interdisciplinary weight loss therapy in bone turnover markers and inflammation biomarkers of obese adolescents

Variables	Baseline	1 year	*p* value	Δ value
Beta-CTx collagen (ng/mL)	0.77 ± 0.46	0.64 ± 0.18	0.26	-0.16 ± 0.54
Osteocalcin (ng/ml)	30.3 (19/57.20)	34.60 (23.10/56.20)	0.28	3.60 (-7.2/43.3)
Adiponectin (µg/L)	1.9 ± 1.06	3.2 ± 1.2*	< 0.001	1.3 ± 1.4
Leptin (ng/ml)	24.65 ± 13.12	24.66 ± 14.53	0.50	-1.16 ± 8.53
Adiponectin/leptin ratio	0.09 ± 0.06	0.17 ± 0.16*	< 0.001	0.08 ± 0.13
IL-15 (pg/mL)	0.05 (0/1.23)	0.10 (0/1.28)*	0.04	0.06 (-0.98/0.81)

Beta-CTx collagen: C-terminal telopeptides of type I collagen; IL-15: interleukin-15.

* Statistical difference p ≤ 0.05. Effects of therapy: comparison between baseline and 1 year of therapy.

### Correlations analysis

#### Baseline values

Negative correlations were showed between bone mineral content (g) with body fat mass (%), visceral fat (cm) with adiponectin (ng/mL) and Beta CTX-collagen (ng/ml) with adiponectin (ng/mL). Positive correlations were demonstrated between bone mineral content (g) with body lean mass (% and kg), visceral fat (cm) with body mass index (kg/m²) and body fat mass (kg), leptin (ng/mL) with body mass index (kg/m²) and body fat mass (kg) (Table 3).

**Table 3:** Correlations analysis

Variables		r	*p* value
Baselines values			
Bone mineral content (g)			
	Body fat mass (%)	-0.48	0.012
	Body lean mass (%)	0.48	0.012
	Body lean mass (kg)	0.56	0.003
Visceral fat (cm)			
	Body mass index (kg/m²)	0.64	0.0001
	Body fat mass (kg)	0.61	0.001
	Adiponectin (ng/mL)	-0.47	0.013
Adiponectin (ng/mL)			
	Beta CTX-collagen (ng/mL)	-0.45	0.03
Leptin (ng/mL)			
	Body mass index (kg/m²)	0.50	0.009
	Body fat mass (kg)	0.46	0.016
**Final values**			
Bone mineral content (g)			
	Body fat mass (%)	-0.47	0.037
	Body lean mass (%)	0.47	0.037
	Body lean mass (kg)	0.51	0.020
Adiponectin/leptin (ng/mL)			
	Subcutaneous fat (cm)	-0.53	0.01
Osteocalcin (ng/mL)			
	Leptin (ng/mL)	-0.52	0.020

#### Final values

Negative correlations were showed between bone mineral content (g) with body fat mass (%); osteocalcin (ng/mL) with leptin (ng/mL); and adiponectin/leptin ratio with subcutaneous fat (cm). Positive correlations were demonstrated between bone mineral content (g) with body lean mass (% and kg) (Table 3).

#### Regression analysis

**Figure 2:**
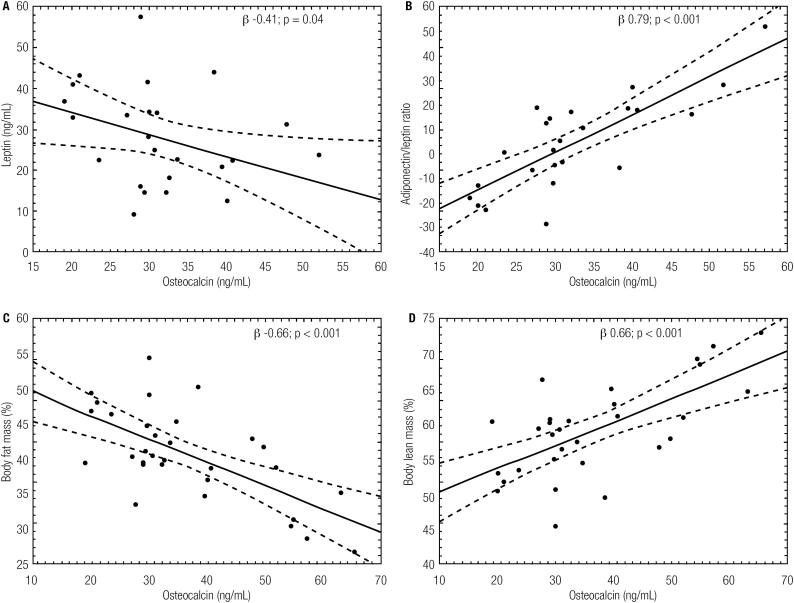
Simple linear regression established between osteocalcin (ng/mL) with: A) leptin (ng/mL); B) adiponectin/leptin ratio; C) body fat mass (%) and D) body lean mass (%).

As shown in the Figure 2 leptin (β −0.41; p = 0.04), adiponectin/leptin ratio (β 0.79; p < 0.001), body fat mass (β −0.66; p < 0.001) and body lean mass (β 0.66; p < 0.001) were predictors for changes in osteocalcin concentration.

## DISCUSSION

The first aim of the present investigation was to analyze the role of inflammatory biomarkers in bone turnover. The most important findings were the negative association with leptin concentration; and the positive correlation between the adiponectin/leptin ratio and osteocalcin. Therefore, we were able to confirm the hypothesis that pro/anti-inflammatory adipokine is a key mediator of bone turnover markers in obese adolescents.

According to our understanding, our group was the first to demonstrate the positive correlation between the adiponectin/leptin ratio and osteocalcin in obese adolescents, showing the beneficial effect of an improved adiponectin/leptin ratio on bone metabolism. Osteocalcin is a biomarker only secreted by osteoblasts, which enables the recognition of cell turnover in the skeletal system (7). Leptin concentration has an important influence on bone metabolism. In an experimental study leptin-deficient ob/ob and leptin-resistant db/db showed increased osteocalcin levels (22), indicating that leptin is an inhibitor of osteoblastic bone formation.

Corroborating with this data, we were able to show a negative correlation between leptin and osteocalcin. In accordance, recently, investigations have shown that a reduction in leptin concentration correlated with an improvement of osteocalcin levels in obese adults (7). Additionally, weight loss promotes an increase in the adiponectin concentration, which plays an important role in bone mass. Another investigation showed that an increase in adiponectin concentration was associated with an improvement in the osteoblastic activity, suggesting an increase in osteocalcin concentration only in the experimental study. However, these authors suggest that adiponectin exerts an activity that increases bone mass by suppressing osteoclastogenesis and by activating osteoblastogenesis, indicating that adiponectin manipulation could be therapeutically beneficial for patients with osteopenia (8).

It is relevant to observed that, in our results a positive correlation was demonstrated between the adiponectin/leptin ratio and osteocalcin in obese adolescents, considering the baseline values. Additionally, at the end of treatment an increase in the adiponectin/leptin ratio without significant statistical changes in osteocalcin was observed. However, it is important to note a discreet change in the osteocalcin concentration was observed in the median baseline values compared with the final value (increase of 12%). In this way, it is possible to suggest that a positive change in the adiponectin/leptin ratio could promote benefits in osteocalcin concentration in obese adolescents. Moreover, a previous study considered the adiponectin/leptin ratio to be a better inflammatory marker for the metabolic syndrome population. This ratio has high sensitivity and specificity for the diagnosis of metabolic syndrome (11).

In fact, in the present study adiponectin was negatively correlated with visceral fat; and adiponectin/leptin correlated negatively with subcutaneous fat. It is likely that the impaired actions of adiponectin are clinically important in obese patients because adiponectin is the most abundant adipocyte-derived hormone with established anti-inflammatory effect (1).

Additionally, in the present investigation, it was found that both lean and body fat mass are predictors of osteocalcin. Concomitant to this, negative and positive correlations were observed between bone mineral content and body fat and lean body mass, respectively. In a previous investigation, it was demonstrated that the serum level of total osteocalcin was positively associated with fat-free mass independent of age, fat mass, leptin, and other confounders in premenopausal women. The hypothesis suggested to explain this association is based on the fact that fat-free mass could indirectly reflect the mechanical load on the bone, which can further stimulate bone formation, whereas low skeletal muscle mass is considered a risk factor for low bone mineral density (23). Moreover, corroborating with our findings, an inverse association was observed between osteocalcin and body fat mass in men with type 2 diabetes and in the elderly population. It is suggested that body fat mass accumulation is closely linked to bone turnover. Adipocytes are responsible for secreting many pro/anti-inflammatory cytokines such as TNF-alpha, interleukin-6, interleukin-10, leptin and adiponectin, which are capable of modulating bone metabolism (24,25).

Notably, Beta-CTx collagen correlates negatively with adiponectin and positively with visceral fat. Adiponectin, an anti-inflammatory adipokine, could promote the acceleration of osteogenesis (26). Beta-CTx collagen is the C-terminal telopeptide of type I collagen, the main component (approximately 90%) of the protein matrix of bone. Beta-CTx collagen is released into the bloodstream during bone resorption and is almost entirely excreted by the kidneys. Its quantification serves as a specific marker for the degradation of mature type I collagen from the bone (5).

In addition, visceral fat promotes a secretion of many pro-inflammatory adipokines that are associated with an increase in the bone reabsorption and a decrease in bone formation (26). In this regard, our results showed that the pro-inflammatory profile present in obese adolescents is associated with an increase in Beta CTX-collagen and weight loss. A reduction in visceral fat, specifically, is associated with an increase in the adiponectin concentration and a reduction in the bone reabsorption biomarker as shown in the correlation analysis.

The second objective was to analyze the effects of interdisciplinary weight loss treatments on pro/anti-inflammatory adipokines and their role in bone turnover markers in obese adolescents. Therefore, another important finding from the present investigation is that weight loss therapy promotes a significant reduction in visceral fat (cm), subcutaneous fat (cm), visceral/subcutaneous ratio, total body fat mass and an increase in the lean body mass enhanced by nutritional counseling and physical exercise training. These results are important since it is well-established in the literature that fat deposition in the visceral compartment is related to the development of some diseases (1,27).

In fact, visceral fat plays a pathological role, due to the secretion of certain pro-inflammatory adipokines also related to the deterioration of bone mass and is associated with development of many comorbidities, such as metabolic syndrome, dyslipidemia, and cardiovascular complications. A prior study showed that an increase of 1 cm in visceral fat was associated with a 1.97 fold (95% CI 1.06–3.66) in boys and 2.08 fold (95% CI 1.38–3.13) in girls increased risk of developing nonalcoholic fatty liver disease (27).

In addition to these results, an increase in the IL-15 concentration after weight loss therapy was observed. IL-15 is considered an important fat mass regulator. In a prior investigation, a negative association between plasma IL-15 and fat mass was found, independent of the diagnosis of type 2 diabetes, which suggests that IL-15 may be involved in the regulation of body fat mass (28). In experimental research, it was found that the administration of IL-15 seems to decrease circulating triglycerides by decreasing both the liver lipogenic rate and very-low-density lipoprotein (VLDL). Moreover, IL-15 decreases lipoprotein lipase activity and the lipogenic rate in adipose tissue. Experimental studies showed a significant decrease in the intestinal lipid absorption, which may in part explain the anti-obesity effects of IL-15. Finally, it has been shown that lipoaspirate-derived human adipocytes treated with IL-15 inhibited pre-adipocyte differentiation. The mechanism of IL-15 signaling in adipocytes however, is currently unknown (29). Together, these findings suggest that IL-15 treatment could result in weight loss and decreased visceral fat, corroborating with the control of the inflammatory state related to obesity (30).

Corroborating with our findings, Brunelli and cols. (31), showed that 24 weeks of moderate-high-intensity combined training (including aerobic and resistance exercises), in obese middle-aged men promotes an increase in the IL-15, concomitant with an improvement in the adiponectin concentration; and a decrease in the body fat. Also, it is suggested that the increase in the fat free mass and decreased body fat mass, may have contributed to the in IL-15 concentration in humans has a relevant anti-inflammatory function in the metabolic profile and may enhance energy expenditure to protect the body from obesity and type 2 diabetes (32).

Additionally, an increase in adiponectin concentration and adiponectin/leptin ratio was observed after interdisciplinary weight loss therapy. Adiponectin is a potent anti-inflammatory adipokine that possesses multiple beneficial effects on obesity-related medical complications (33). It may also have anti-atherogenic and anti-inflammatory properties, and high levels of circulating adiponectin have been related to a lower risk of coronary heart disease (34). However, no statistical difference was observed in the leptin concentration, probably explained because at baseline the adolescents showed a normal leptin concentration as recommended for this population (22). Although, it is relevant to note that hyperleptinemia is consider an important condition that contribute to the pro-inflammatory state observed in obesity population, including adolescents, and associated with comorbidities development (35). Considering bone health, studies demonstrated a negatively association between leptin and bone turnover biomarkers, especially with osteocalcin (7,10,36,37).

Another important result was the increase in BMC. This finding is possibly associated with improvement in the inflammatory profile, a reduction in visceral fat and the benefits of physical exercise intervention combined with other therapies realized by volunteers during the development of the study. It has been previously demonstrated that physical exercise can improve osteogenesis, which consequently increases bone mineral density and content (38). Moreover, resistance physical exercise at moderate intensity is related to decreased bone resorption markers (39). We know that aerobic physical exercises are associated with weight loss, but the combination of two kinds of physical exercise in the same session (aerobic plus resistance training) could optimize weight loss with greater benefits such as improvement in bone turnover markers and lean body mass as previously shown (2,40) as well as in the present study.

Finally, we showed that interdisciplinary weight loss therapy improves pro/anti-inflammatory profile and was related to bone turnover. Together, our results suggest the importance of controlling the inflammatory state and the effects on bone turnover markers related to obesity in adolescence.

## Conclusions and futures directions

In the present study, we were able to show that both leptin and adiponectin/leptin ratio were negatively and positively associated with osteocalcin, respectively, modulating bone turnover markers. Finally, the interdisciplinary weight loss treatments were seen to be effective at reducing body fat mass, visceral fat and at increasing lean body mass, bone mineral content, adiponectin and IL-15. Together, these results suggest that this kind of intervention is considered an interesting alternative to prevent and treat obesity and promote bone health.

## References

[1] Dâmaso AR, de Piano A, Campos RMS, Corgosinho FC, Siegfried W, Caranti DA, et al. Multidisciplinary approach to the treatment of obese adolescents: effects on cardiovascular risk factors, inflammatory profile, and neuroendocrine regulation of energy balance. Int J Endocrinol. 2013;2013:541032. 10.1155/2013/541032 PMC3826292 24285955

[2] Campos RM, de Mello MT, Tock L, da Silva PL, Corgosinho FC, Carnier J, et al. Interaction of bone mineral density, adipokines and hormones in obese adolescents girls submitted in an interdisciplinary therapy. J Pediatr Endocrinol Metab. 2013;26(7-8):663-8. 10.1515/jpem-2012-0336 23612645

[3] Campos RM, Lazaretti-Castro M, Mello MT, Tock L, Silva PL, Corgosinho FC, et al. Influence of visceral and subcutaneous fat in bone mineral density of obese adolescents. Arq Bras Endocrinol Metabol. 2012;56:12-8. 10.1590/s0004-27302012000100003 22460190

[4] Lac G, Cavalie H, Ebal E, Michaux O. Effects of a high fat diet on bone of growing rats. Correlations between visceral fat, adiponectin and bone mass density. Lipids Health Dis. 2008;7:16. 10.1186/1476-511X-7-16 PMC2386795 18442361

[5] Peichl P, Griesmacherb A, Marteau R, Hejc S, Kumpan W, Müller MM, et al. Serum crosslaps in comparison to serum osteocalcin and urinary bone resorption markers. Clin Biochem. 2001;34(2):131-9. 10.1016/s0009-9120(01)00193-x 11311223

[6] Alfadda AA, Masood A, Shaik SA, Dekhil H, Goran M. Association between osteocalcin, metabolic syndrome, and cardiovascular risk factors: role of total and undercarboxylated osteocalcin in patients with type 2 diabetes. Int J Endocrinol. 2013;2013:197519. 10.1155/2013/197519 PMC3638647 23653641

[7] Suh HS, Hwang IC, Lee KS, Kim KK. Relationships between serum osteocalcin, leptin and the effect of weight loss by pharmacological treatment in healthy, nonsmoking Korean obese adults. Clin Chim Acta. 2013;418:17-21. 10.1016/j.cca.2012.11.029 23247052

[8] Oshima K, Nampei A, Matsuda M, Iwaki M, Fukuhara A, Hashimoto J, et al. Adiponectin increases bone mass by suppressing osteoclast and activating osteoblast. Biochem Biophys Res Commun. 2005;331(2):520-6. 10.1016/j.bbrc.2005.03.210 15850790

[9] Kanazawa I, Yamaguchi T, Yano S, Yamauchi M, Yamamoto M, Sugimoto T. Adiponectin and AMP kinase activator stimulate proliferation, differentiation, and mineralization of osteoblastic MC3T3-E1 cells. BMC Cell Biol. 2007;8:51. 10.1186/1471-2121-8-51 PMC2214728 18047638

[10] Saleem U, Mosley TH Jr, Kullo IJ. Serum osteocalcin is associated with measures of insulin resistance, adipokine levels, and the presence of metabolic syndrome. Arterioscler Thromb Vasc Biol. 2010;30(7):1474-8. 10.1161/ATVBAHA.110.204859 PMC2939910 20395593

[11] Mirza S, Qu HQ, Li Q, Martinez PJ, Rentfro AR, McCormick JB, et al. Adiponectin/leptin ratio and metabolic syndrome in a Mexican American population. Clin Invest Med. 2011;34(5):E290. 10.25011/cim.v34i5.15672 PMC3256930 21968271

[12] Choi BC, Pak AW. Multidisciplinarity, interdisciplinarity and transdisciplinarity in health research, services, education and policy: 1. Definitions, objectives, and evidence of effectiveness. Clin Invest Med. 2006;29(6):351-64. 17330451

[13] Tanner JM, Whitehouse RH. Clinical longitudinal standards for height, weight, height velocity, weight velocity, and stages of puberty. Arch Dis Child. 1976;51(3):170-9. 10.1136/adc.51.3.170 PMC1545912 952550

[14] Centers for Disease Control and Prevention. Hyattsville: National Center for Health Statistics. (Updates on 11 January 2007). Prevalence of overweight among children and adolescents: United States 1999-2002. Disponível em: http://www.cdc.gov/nchs/products/pubs/pubd/hestats/overwght99.htm. Acesso em: 11 ago. 2013.

[15] Black E, Petersen L, Kreutzer M, Toubro S, Sørensen TI, Pedersen O, et al. Fat mass measured by DXA varies with scan velocity. Obes Res. 2002;10(2):69-77. 10.1038/oby.2002.12 11836451

[16] Dossus L, Becker S, Achaintre D, Kaaks R, Rinaldi S. Validity of multiplex-based assays for cytokine measurements in serum and plasma from “non-diseased” subjects: comparison with ELISA. J Immunol Methods. 2009;350(1-2):125-32. 10.1016/j.jim.2009.09.001 19748508

[17] Gutin B, Ramsey L, Barbeau P, Cannady W, Ferguson M, Litaker M, et al. Plasma leptin concentrations in obese children: changes during 4-mo periods with and without physical training. Am J Clin Nutr. 1999;69(3):388-94. 10.1093/ajcn/69.3.388 10075321

[18] Ribeiro-Filho FF, Faria AN, Azjen S, Zanella MT, Ferreira SR. Methods of estimation of visceral fat: advantages of ultrasonography. Obes Res. 2003;11(12):1488-94. 10.1038/oby.2003.199 14694213

[19] Donnelly JE, Blair SN, Jakicic JM, Manore MM, Rankin JW, Smith BK; American College of Sports Medicine. American College of Sports Medicine Position Stand. Appropriate physical activity intervention strategies for weight loss and prevention of weight regain for adults. Med Sci Sports Exerc. 2009;41(2):459-71. 10.1249/MSS.0b013e3181949333 19127177

[20] Kraemer WJ, Ratamess NA, French DN. Resistance training for health and performance. Curr Sports Med Rep. 2002;1(3):165-71. 10.1249/00149619-200206000-00007 12831709

[21] National Academic Press. Dietary Reference Intake. Applications in Dietary Assessment. Washington, DC; 2001.

[22] Ducy P, Amling M, Takeda S, Priemel M, Schilling AF, Beil FT, et al. Leptin inhibits bone formation through a hypothalamic relay: a central control of bone mass. Cell. 2000;100(2):197-207. 10.1016/s0092-8674(00)81558-5 10660043

[23] Wu CH, Yang KC, Chang HH, Yen JF, Tsai KS, Huang KC. Sarcopenia is related to increased risk for low bone mineral density. J Clin Densitom. 2013;16(1):98-103. 10.1016/j.jocd.2012.07.010 22975297

[24] Kanazawa I, Yamaguchi T, Yamauchi M, Yamamoto M, Kurioka S, Yano S, et al. Serum undercarboxylated osteocalcin was inversely associated with plasma glucose level and fat mass in type 2 diabetes mellitus. Osteoporos Int. 2011;22(1):187-94. 10.1007/s00198-010-1184-7 20165834

[25] Kindblom JM, Ohlsson C, Ljunggren O, Karlsson MK, Tivesten A, Smith U, et al. Plasma osteocalcin is inversely related to fat mass and plasma glucose in elderly Swedish men. J Bone Miner Res. 2009;24(5):785-91. 10.1359/jbmr.081234 19063687

[26] Lee HW, Kim SY, Kim AY, Lee EJ, Choi JY, Kim JB. Adiponectin stimulates osteoblast differentiation through induction of COX2 in mesenchymal progenitor cells. Stem Cells. 2009;27(9):2254-62. 10.1002/stem.144 19522015

[27] Dâmaso AR, do Prado WL, de Piano A, Tock L, Caranti DA, Lofrano MC, et al. Relationship between nonalcoholic fatty liver disease prevalence and visceral fat in obese adolescents. Dig Liver Dis. 2008;40(2):132-9. 10.1016/j.dld.2007.09.009 18082476

[28] Nielsen AR, Hojman P, Erikstrup C, Fischer CP, Plomgaard P, Mounier R, et al. Association between interleukin-15 and obesity: interleukin-15 as a potential regulator of fat mass. J Clin Endocrinol Metab. 2008;93(11):4486-93. 10.1210/jc.2007-2561 18697873

[29] Almendro V, Carbó N, Busquets S, López-Soriano J, Figueras M, Ametller E, et al. Interleukin-15 decreases lipid intestinal absorption. Int J Mol Med. 2005;15(6):963-7. 15870900

[30] Carbó N, López-Soriano J, Costelli P, Alvarez B, Busquets S, Baccino FM, et al. Interleukin-15 mediates reciprocal regulation of adipose and muscle mass: a potential role in body weight control. Biochim Biophys Acta. 2001;1526(1):17-24. 10.1016/s0304-4165(00)00188-4 11287118

[31] Brunelli DT, Chacon-Mikahil MP, Gáspari AF, Lopes WA, Bonganha V, Bonfante IL, et al. Combined training reduces subclinical inflammation in obese middle-age men. Med Sci Sports Exerc. 2015;47(10):2207-15. 10.1249/MSS.0000000000000658 26378946

[32] Ye J. Beneficial metabolic activities of inflammatory cytokine interleukin 15 in obesity and type 2 diabetes. Front Med. 2015;9(2):139-45. 10.1007/s11684-015-0377-z PMC4559335 25511621

[33] Manigrasso MR, Ferroni P, Santilli F, Taraborelli T, Guagnano MT, Michetti N, et al. Association between circulating adiponectin and interleukin-10 levels in android obesity: effects of weight loss. J Clin Endocrinol Metab. 2005;90(10):5876-9. 10.1210/jc.2005-0281 16030165

[34] Wang Y, Zhou M, Lam KS, Xu A. Protective roles of adiponectin in obesity-related fatty liver diseases: mechanisms and therapeutic implications. Arq Bras Endocrinol Metabol. 2009;53(2):201-12. 10.1590/s0004-27302009000200012 19466213

[35] Dâmaso AR, de Piano A, Sanches PL, Corgosinho F, Tock L, Oyama LM, et al. Hyperleptinemia in obese adolescents deregulates neuropeptides during weight loss. Peptides. 2011;32(7):1384-91. 10.1016/j.peptides.2011.04.025 21641948

[36] Giudici KV, Kindler JM, Martin BR, Laing EM, McCabe GP, McCabe LD, et al. Associations among osteocalcin, leptin and metabolic health in children ages 9-13 years in the United States. Nutr Metab (Lond). 2017;14:25. 10.1186/s12986-017-0171-9 PMC5341348 28286536

[37] Jürimäe J, Lätt E, Mäestu J, Saar M, Purge P, Maasalu K, et al. Osteocalcin is inversely associated with adiposity and leptin in adolescent boys. J Pediatr Endocrinol Metab. 2015;28(5-6):571-7. 10.1515/jpem-2014-0432 25741787

[38] Lanyon LE, Rubin CT. Static vs dynamic loads as an influence on bone remodelling. J Biomech. 1984;17(12):897-905. 10.1016/0021-9290(84)90003-4 6520138

[39] Whipple TJ, Le BH, Demers LM, Chinchilli VM, Petit MA, Sharkey N, et al. Acute effects of moderate intensity resistance exercise on bone cell activity. Int J Sports Med. 2004;25(7):496-501. 10.1055/s-2004-820942 15459829

[40] Foschini D, Araújo RC, Bacurau RF, De Piano A, De Almeida SS, Carnier J, et al. Treatment of obese adolescents: the influence of periodization models and ACE genotype. Obesity (Silver Spring). 2010;18(4):766-72. 10.1038/oby.2009.247 19680237

